# Association between systolic blood pressure and dementia in the Whitehall II cohort study: role of age, duration, and threshold used to define hypertension

**DOI:** 10.1093/eurheartj/ehy288

**Published:** 2018-06-12

**Authors:** Jessica G Abell, Mika Kivimäki, Aline Dugravot, Adam G Tabak, Aurore Fayosse, Martin Shipley, Séverine Sabia, Archana Singh-Manoux

**Affiliations:** 1 INSERM, U1018, Centre for Research in Epidemiology and Population Health, Université Paris-Saclay, Hôpital Paul Brousse, Bât 15/16, 16 Avenue Paul Vaillant Couturier, 94807 Villejuif Cedex, France; 2 Department of Epidemiology and Public Health, University College London, London, UK; 3 Faculty of Medicine, 1st Department of Medicine, Semmelweis University, Budapest, Hungary

**Keywords:** Blood pressure, Dementia, Ageing

## Abstract

**Aims:**

To examine associations of diastolic and systolic blood pressure (SBP) at age 50, 60, and 70 years with incidence of dementia, and whether cardiovascular disease (CVD) over the follow-up mediates this association.

**Methods and results:**

Systolic and diastolic blood pressure were measured on 8639 persons (32.5% women) from the Whitehall II cohort study in 1985, 1991, 1997, and 2003. Incidence of dementia (*n* dementia/*n* total = 385/8639) was ascertained from electronic health records followed-up until 2017. Cubic splines using continuous blood pressure measures suggested SBP ≥130 mmHg at age 50 but not at age 60 or 70 was associated with increased risk of dementia, confirmed in Cox regression analyses adjusted for sociodemographic factors, health behaviours, and time varying chronic conditions [hazard ratio (HR) 1.38; 95% confidence interval (95% CI) 1.11, 1.70]. Diastolic blood pressure was not associated with dementia. Participants with longer exposure to hypertension (SBP ≥ 130 mmHg) between mean ages of 45 and 61 years had an increased risk of dementia compared to those with no or low exposure to hypertension (HR 1.29, 95% CI 1.00, 1.66). In multi-state models, SBP ≥ 130 mmHg at 50 years of age was associated with greater risk of dementia in those free of CVD over the follow-up (HR 1.47, 95% CI 1.15, 1.87).

**Conclusion:**

Systolic blood pressure ≥130 mmHg at age 50, below the conventional ≥140 mmHg threshold used to define hypertension, is associated with increased risk of dementia; in these persons this excess risk is independent of CVD.

## Introduction

Randomized trials on older adults show no effect of lowering of blood pressure on the risk of dementia.^1^^,^^2^ Age is known to modify the association between hypertension and dementia.^3–6^ Observational data suggest that hypertension in mid-life increases the risk of dementia in later life,^7–9^ leading it to be included as a putative risk factor in dementia prevention guidelines.^10^ Nonetheless, there are several outstanding questions. One, ‘mid-life’ remains poorly characterized in studies, ranging from 35 to 68 years.^8^^,^^11–13^ Two, few studies have attempted to assess the impact of duration of hypertension using measures of blood pressure rather than reported use of antihypertensive medication. Three, it is unclear whether the 140 mmHg systolic blood pressure (SBP) threshold to define hypertension^14^ in mid-life is appropriate for dementia risk, given age-specific treatment targets recommended in recent reports aimed at reducing cardiovascular disease (CVD).^15^^,^^16^

To address some of these limitations, we examined the association between measured SBP and diastolic blood pressure (DBP) and dementia, focusing on the effects of age, duration of high blood pressure, and threshold of blood pressure to define hypertension in a large prospective cohort followed for 30 years. We hypothesize that hypertension in mid-life (at age 50) but not at older ages (age 70), is associated with risk of dementia. Given evidence of silent strokes and white matter lesions in those with hypertension,^3^ a second hypothesis we tested is that the association between hypertension at age 50 and incidence of dementia is only partially explained by clinical CVD over the follow-up.

## Methods

The Whitehall II study is an ongoing study of 6895 men and 3413 women, aged 35–55 in 1985; follow-ups examinations were conducted in 1991 (*n* = 8815), 1997 (*n* = 7870), 2003 (*n* = 6967), 2007 (*n* = 6967), 2012 (*n* = 6318), and 2015 (*n* = 5632) with each wave taking 2 years to complete. Written informed consent from participants and research ethics approvals were renewed at each contact; the most recent approval was from the University College London Hospital Committee on the Ethics of Human Research, reference number 85/0938.

### Blood pressure

Systolic and diastolic blood pressure were measured in the sitting position after 5 min of rest, using the Hawksley random-zero sphygmomanometer in 1985, 1991, and 1997 and an OMRON HEM 907 digital sphygmomanometer in 2003, 2007, and 2012. At each wave, two measures of SBP and DBP were taken and their mean was used in the analysis. Use of antihypertensive medication was self-reported at each contact.

### Dementia

A comprehensive tracing of electronic health records, involving three databases, was used for dementia ascertainment: hospital episode statistics (HES) database, the Mental Health Services Data Set (MHSDS), and the mortality register using The International Classification of Disease, Tenth Revision (ICD-10) codes (F00–F04, G30, and G31), record linkage until 31 March 2017. These are national databases: HES and MHSDS contain information on both in- and out-patient care, with the latter also including data on care in the community. The validity of dementia cases in our study is supported by modelling changes in the global cognitive score in the 10 years before dementia diagnosis (Supplementary material online, *Figure S1*) as in studies that use a ‘gold-standard’ dementia ascertainment procedure.^17^

### Covariates

*Sociodemographic factors* included age, sex, ethnicity (white, non-white), education, and occupational position.

*Health behaviours* included smoking status (current, ex-, and never-smoker); alcohol consumption: *non-drinkers*, *moderate alcohol consumption* (1–14 units/week in women or 1–21 units/week in men), and *heavy alcohol consumption* (>14 units/week in women or >21 units/week in men), one unit of alcohol (UK) is defined as 10 ml (8 g) of pure alcohol; physical activity (h/week of moderate or vigorous physical activity, categorized as *low* <1 h, *moderate* between 1 and 2.5 h and *high* ≥2.5 h); and frequency of fruit and vegetable consumed per week (<once/day, once/day, or >once/day).

*Health status.* Body mass index (BMI, kg/m^2^) was calculated from measured height and weight. *Diabetes* was determined by fasting glucose ≥7.0 mmol/l, a 2-h post-load glucose ≥11.1 mmol/l, doctor-diagnosed diabetes, or use of diabetes medication. *Coronary heart disease* (CHD) by study specific assessments (12-lead resting electrocardiogram (ECG) recording, coded using the Minnesota system), self-reported CHD (verified in medical records), and linkage to HES (The International Classification of Disease, Ninth Revision (ICD-9) codes 410–414, ICD10 codes I20–I25, or procedures K40–K49, K50, K75, U19). *Stroke* using the MONICA-Ausburg stroke questionnaire, corroborated in HES (ICD9 430, 431, 434, 436 and ICD10 I60–I64). *Atrial fibrillation* assessment was based on data from a 12-lead resting ECG (Mingorec, Siemens Healthcare, Erlangen, Germany), Minnesota code 8.3 and data from HES (ICD9 code 427.3 and ICD10 code I48). *Heart failure*, based on HES records (ICD10 code I50). Use of medication for CVD was self-reported.

### Statistical analysis

We extracted data on blood pressure at ages 50, 60, and 70 years for each participant across the data waves, allowing a ±5 year margin for each age category. The analysis of blood pressure at age 50, 60, and 70 years was based on 8639, 7558, and 4989 participants, respectively: the numbers differ due to non-response, death before age 60 or 70 or participants not having reached 70 years at the end of follow-up. We used inverse probability weighting (IPW)^18^ to ensure that analyses at ages 50, 60, and 70 reflected the same set of individuals. The probability of remaining in the study sample was estimated using data on sociodemographic, behavioural, cardiometabolic risk factors, chronic conditions, antihypertensive medication, and dementia status including its interaction with SBP and DBP and antihypertensive medication. The inverse of these probabilities were used to weight the data in Cox regression. The analyses, described below, were undertaken using STATA 14.1; the null hypothesis was rejected for two-sided values of *P* < 0.05.

#### Age and threshold of blood pressure and incidence of dementia

We used Cox regression with separate models for age 50, 60, and 70 years; the proportional hazard assumption was verified using Schoenfeld residuals. Date of entry was the date of clinical assessment from which the hypertension measure was drawn. Participants were censored at record of dementia, death, or 31 March 2017, whichever occurred first. We first examined the threshold of blood pressure at ages 50, 60, and 70 using restricted cubic spline regressions with Harrell knots^19^; the command *xblc*^20^ was used to estimate adjusted HRs for the association of blood pressure (continuous measure in mmHg) with the risk of dementia. Once the threshold was identified, we used a dichotomous definition of hypertension to assess associations with dementia. The basic analysis was adjusted for sociodemographic (Model 1), then behavioural (Model 2), and finally also health-related factors (Model 3).

#### Duration of hypertension and incidence of dementia

Duration of hypertension status was estimated using data from 1985, 1991, 1997, and 2003 (mean age of participants 44.9 years in 1985 and 61.1 years in 2003) on 8313 participants who were alive and free of dementia in 2003 and had at least two assessments of hypertension status. Duration was summarized using group based trajectory modelling, fitted using the command *traj* in STATA. The association between hypertension trajectories (low, increasing, and high) and subsequent incidence of dementia was examined using Cox regression, with age as the time-scale. Participants were censored at date of record of dementia, death, or 31 March 2017, whichever occurred first. The covariates in Models 1, 2, and 3 were as in the previous analyses and IPW was used to account for missing data.

#### Role of cardiovascular disease in the association between hypertension at age 50 and dementia

We examined the mediating role of CVD (stroke and CHD) over the follow-up in the association between hypertension and incidence of dementia using multi-state models with a Weibull distribution. These models are an extension of competing risks survival analysis, allowing simultaneous estimation of the risk associated with hypertension in (i) the incidence of CVD, (ii) the risk of dementia in those with CVD, and (iii) the risk of dementia in those free of CVD. Age was used as the timescale, and models were adjusted for sociodemographic factors. These analyses were undertaken using R (mstate).

## Results

Characteristics of 8639 participants at age 50 are presented in *Table 1* as a function of dementia status over the follow-up. Mean age at dementia diagnosis was 75.2 (standard deviation = 5.4) years. Incidence of dementia was associated with lower education, higher blood pressure, and co-morbidities.

**Table 1:** Participant characteristics at age 50 by dementia status at the end of follow-up^a^

	Overall (*n* = 8639)	No dementia (*n* = 8254)	Dementia (*n* = 385)	*P*-value^b^
Characteristics at age 50				
Female, *n* (%)	2811 (32.5)	2642 (32.0)	169 (43.9)	<0.001
Non-white ethnicity, *n* (%)	916 (10.6)	855 (10.4)	61 (15.8)	<0.001
No educational qualifications, *n* (%)	913 (10.6)	845 (10.2)	68 (17.7)	<0.001
Not married/cohabiting, *n* (%)	2108 (24.4)	1996 (24.2)	112 (26.1)	0.028
Low occupational position, *n* (%)	1695 (19.6)	1551 (18.8)	144 (37.4)	<0.001
BMI, M (SD)	25.5 (3.8)	25.5 (3.8)	25.9 (4.0)	0.022
Current smokers, *n* (%)	1345 (15.6)	1265 (15.3)	80 (20.8)	0.004
Heavy alcohol consumption^c^, *n* (%)	1643 (19.0)	1587 (19.2)	56 (14.6)	<0.001
Poor diet^d^, *n* (%)	3285 (38.0)	3117 (37.8)	168 (43.6)	0.003
Low physical activity, *n* (%)	2000 (23.2)	1884 (22.8)	116 (30.1)	0.003
Diabetes, *n* (%)	193 (2.2)	180 (2.2)	13 (3.4)	0.121
Systolic blood pressure, M (SD)	122.1 (14.9)	121.9 (14.8)	126.3 (16.6)	<0.001
Diastolic blood pressure, M (SD)	78.5 (10.3)	78.4 (10.3)	80.0 (10.8)	0.003
Antihypertensive medication, *n* (%)	492 (5.7)	467 (5.7)	25 (6.5)	0.49
Cardiovascular disease, *n* (%)	1654 (19.2)	1540 (18.7)	114 (29.6)	<0.001
Atrial fibrillation, *n* (%)	758 (8.8)	698 (8.5)	60 (15.6)	<0.001
Heart failure, *n* (%)	262 (3.0)	236 (2.9)	26 (6.8)	<0.001
Cardiovascular medication^f^, *n* (%)	4564 (52.8)	4366 (52.9)	198 (51.4)	0.57

AF, atrial fibrillation; BMI, body mass index; CVD, cardiovascular disease; HF, heart failure; M, mean; SD, standard deviation.

a Dementia status: start of follow-up at age 50 years, end of follow-up March 2017.

b χ^2^ tests (categorical data) and analysis of variance (continuous data).

c Heavy alcohol consumption was defined as >14 units/week in women and > 21 units/week in men. ^d^Poor diet was defined as fruit and vegetable consumption < once a day.

e *n* (%) of participants who had experienced CVD, AF or HF from age 50 (baseline) to the end of the follow-up.

f Participants who had *ever* reported taking cardiovascular medication over the follow-up.

### Age and threshold of blood pressure and incidence of dementia

**Figure 1:**
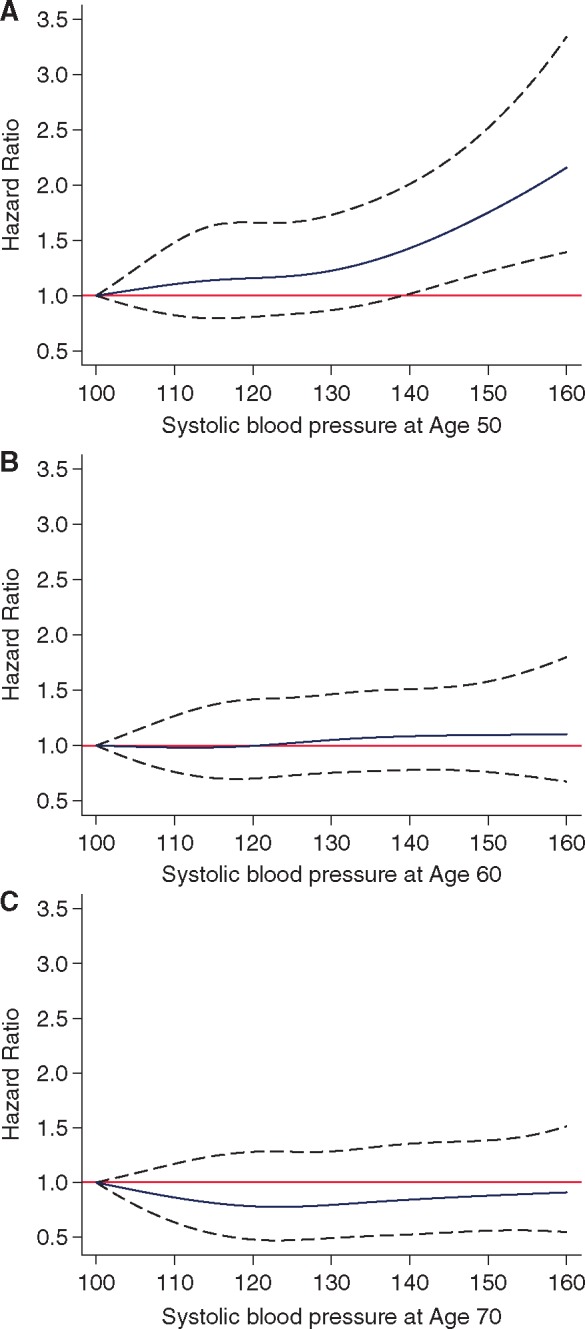
Threshold: association of systolic blood pressure^a,b^ at age 50 (*A*), 60 (*B*), and 70 years (*C*) with dementia. ^a^Systolic blood pressure was modelled by both tail restricted cubic splines with four age-specific Harrell knots in a Cox regression model adjusted for age, sex, education, ethnicity, marital status, and occupational position. ^b^Hazard ratios calculated with systolic blood pressure 100 mmHg as reference.

Continuous measures of blood pressure using cubic splines suggested increased risk of dementia with higher SBP starting from 130 mmHg at age 50 (*Figure **1*, Panel A). In contrast, no association was seen with SBP at age 60 (*Figure 1*, Panel B) or age 70 (*Figure 1*, Panel C). In order to ensure results were robust to overfitting we categorized SBP (<110, 110–119, 120–129, 130–139, ≥140) and obtained similar results (Supplementary material online, *Table S1*). There was no association between DBP at age 50, 60, or 70 years and incidence of dementia (Supplementary material online, *Figure S2*).

In further analyses using Cox regression, SBP ≥130 mmHg at age 50 was associated with increased hazard of dementia in the fully adjusted model [hazard ratio (HR) 1.38; 95% confidence interval (CI) 1.11, 1.70; *Table 2*]. No association was observed when SBP was measured at ages 60 or 70 years (*Table 2*). Further analysis using DBP threshold ≥90 mmHg in addition to SBP to define hypertension revealed similar results (Supplementary material online, *Table S2*).

**Table 2:** Age and threshold of systolic blood pressure: association between hypertension and incidence of dementia^a^

	*n* dementia /*n* total	Model 1	Model 2	Model 3
		HR (95% CI)	HR (95% CI)	HR (95% CI)
Hypertension at age 50 years (*n* = 8639)				
Systolic blood pressure ≥140 mmHg				
No	311/7586	1.00	1.00	1.00
Yes	74/1053	1.39 (1.08, 1.80)	1.40 (1.09, 1.81)	1.30 (1.00, 1.69)
Systolic blood pressure ≥130 mmHg				
No	228/6166	1.00	1.00	1.00
Yes	157/2473	1.45 (1.18, 1.79)	1.45 (1.18, 1, 78)	1.38 (1.11, 1.70)
Systolic blood pressure ≥120 mmHg				
No	147/4007	1.00	1.00	1.00
Yes	238/4632	1.20 (0.97, 1.48)	1.18 (0.96, 1.46)	1.10 (0.89, 1.37)
Hypertension at age 60 years (*n* = 7558)				
Systolic blood pressure ≥140 mmHg				
No	275/6219	1.00	1.00	1.00
Yes	65/1339	1.16 (0.87, 1.54)	1.15 (0.87, 1.53)	1.15 (0.87, 1.53)
Systolic blood pressure ≥130 mmHg				
No	211/4811	1.00	1.00	1.00
Yes	129/2747	1.05 (0.84, 1.32)	1.06 (0.84, 1.33)	1.03 (0.82, 1.31)
Systolic blood pressure ≥120 mmHg				
No	131/2936	1.00	1.00	1.00
Yes	209/4622	1.07 (0.85, 1.34)	1.08 (0.86, 1.36)	1.06 (0.84, 1.34)
Hypertension at age 70 years (*n* = 4989)				
Systolic blood pressure ≥140 mmHg				
No	176/3722	1.00	1.00	1.00
Yes	69/1267	1.00 (0.73, 1.36)	0.98 (0.71, 1.34)	1.03 (0.74, 1.43)
Systolic blood pressure ≥130 mmHg				
No	121/2694	1.00	1.00	1.00
Yes	124/2295	1.07 (0.80, 1.42)	1.06 (0.80, 1.42)	1.14 (0.85, 1.54)
Systolic blood pressure ≥120 mmHg				
No	69/1463	1.00	1.00	1.00
Yes	176/3526	1.07 (0.78, 1.46)	1.06 (0.78, 1.45)	1.19 (0.86, 1.64)

Model 1: Adjusted for age, sex, education, ethnicity, marital status, and occupational position.

Model 2: Model 1 + health behaviours.

Model 3: Model 2 + BMI, diabetes at start of follow-up + time-dependent cardiovascular disease (coronary heart disease, stroke), atrial fibrillation, heart failure, and cardiovascular medication.

CI, confidence interval; HR, hazard ratio.

a Analysis using inverse probability weighting in Cox regression.

Adding use of antihypertensive medication to SBP to define hypertension yielded results (Supplementary material online, *Table S3*) broadly similar to those presented in *Table 2*. In mutually adjusted models (data not tabulated), SBP ≥130 mmHg at age 50 was associated with risk of dementia (HR 1.37; 95% CI 1.11, 1.70), while use of medication was not (HR 1.16, 95% CI 0.75, 1.80). However, at age 60, SBP ≥130 mmHg was not associated with dementia (HR 1.03, 95% CI 0.81, 1.30) but use of antihypertensives was associated with increased risk (HR 1.62, 95% CI 1.21, 2.18). At age 70, SBP ≥130 mmHg was not associated with dementia (HR 1.14, 95% CI 0.85, 1.54) and neither was use of antihypertensives (HR 1.32, 95% CI 0.96, 1.82).

**Table 3:** Duration of hypertension (systolic blood pressure ≥130 mmHg) trajectories^a^ with incidence of dementia^b^

*n* = 8313		Model 1	Model 2	Model 3
	*n* cases/*n* total	HR (95% CI)	HR (95% CI)	HR (95% CI)
Hypertension trajectories (data from 1985, 1991, 1997, 2003)				
Group 1: Low	135/4054	1.00	1.00	1.00
Group 2: Increasing	65/1545	1.08 (0.80, 1.47)	1.12 (0.82, 1.53)	1.15 (0.84, 1.57)
Group 3: High	158/2714	1.30 (1.02, 1.65)	1.31 (1.02, 1.68)	1.29 (1.00, 1.66)

Model 1: Adjusted for age, sex, education, ethnicity, marital status, and occupational position.

Model 2: Model 1 + health behaviours.

Model 3: Model 2 + BMI, diabetes at start of follow-up + time-dependent cardiovascular disease (coronary heart disease, stroke), atrial fibrillation, heart failure, and cardiovascular medication.

CI, confidence interval; HR, hazard ratio.

a Trajectories over a mean 16 year period; further information in Supplementary material online, *Figure S3* and *Table S4*.

b Analysis using inverse probability weighting in Cox regression.

### Sixteen-year hypertension trajectories (systolic blood pressure ≥130 mmHg) and incidence of dementia

Three hypertension trajectories were identified (model fit statistics in Supplementary material online, *Table S4* and graphical representation in Supplementary material online, *Figure S3*): *low, increasing, and high* trajectories comprising 48.8%, 18.6%, and 32.7% of participants, respectively. *Table 3* shows a higher hazard of dementia (Model 3, HR 1.29, 95% CI 1.00, 1.66) in the *high* trajectory group compared to those with *low* prevalence of hypertension with associations in the *increasing* trajectory group (Model 3, HR 1.15, 95% CI 0.84, 1.57) not statistically significant. In trajectories using SBP ≥ 130 mmHg and antihypertensive medication to define hypertension results were similar (Supplementary material online, *Table S5*).

### Role of cardiovascular disease in the association between hypertension at age 50 and dementia

**Figure 2:**
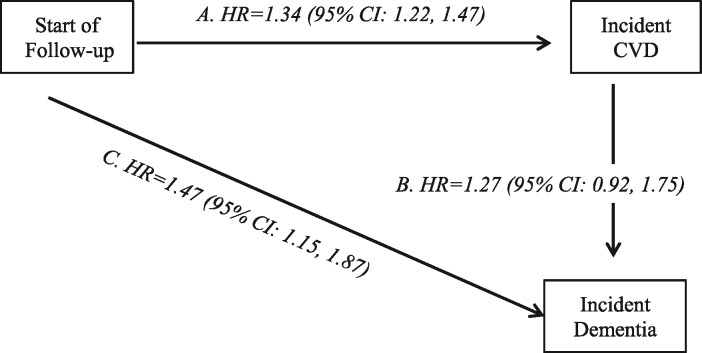
Multi-state models for the role of hypertension at age 50 in transition to cardiovascular disease (stroke or coronary heart disease) and dementia. Role of hypertension (systolic blood pressure ≥130 mmHg) at age 50 years in the risk of transitions from: (*A*) healthy state to incident cardiovascular disease; (*B*) cardiovascular disease (stroke or coronary heart disease) to incident dementia; (*C*) healthy to incident dementia in those free of cardiovascular disease (stroke or coronary heart disease). Analyses with age as timescale and adjusted for sex, education, ethnicity, marital status at age 50, occupation position at age 50, and birth cohort.

*Figure* *2* shows that in multi-state models, hypertension at age 50 was associated with a 1.34 times increased hazard of CVD (95% CI 1.22, 1.47). The association between hypertension at age 50 and dementia was not fully explained by CVD as demonstrated by the increased hazard of dementia in those free of CVD over the follow-up (HR 1.47, 95% CI 1.15, 1.87).

## Discussion

The findings of this longitudinal observational study of over 8000 men and women support the hypothesis that hypertension in mid-life but not late life is associated with increased risk of dementia.^3^^,^^5^ We show that high SBP at age 50 was associated with increased risk of dementia, much under the conventional 140 mmHg threshold used to define hypertension. In our data, the excess risk was apparent at around 130 mmHg of SBP. Hypertension at age 70 was not associated with incidence of dementia. Longer exposure to hypertension in mid-life was associated with increased hazard of dementia. Although adjustment for CVD attenuated associations between hypertension at age 50 and dementia; multi-state models show this association to be present in those free of CVD, suggesting that clinical CVD does not fully account for the association between hypertension and dementia. These findings, highlighting the importance of elevated systolic pressure at age 50 as a risk factor for dementia need to be replicated in larger studies to allow elaboration of evidence based prevention.

The age-specific association between hypertension and dementia is widely acknowledged.^3–5^ Several prospective studies show high blood pressure in mid-life to be associated with increased risk of dementia,^7^^,^^9^^,^^13^ while the evidence of an association between hypertension in late life and dementia is less consistent.^21–27^ The age related variation in the association was shown within a single study in The Adult Changes in Thought Study, where high SBP was associated with dementia in the youngest age group but no association was found in older subjects.^4^ However, previous studies on mid-life hypertension cover a wide range of ages. In the Honolulu Asia Aging study, for example, mid-life was defined as ages between 45 and 68 years, an age-range greater than two decades.^12^ Poor characterization of the age when hypertension carries a risk is also reflected in dementia guidelines as they only use the generic term ‘mid-life’.^10^ Our data show considerable differences in dementia risk over the two decade age-span, elevated SBP at age 50 but not at age 60 increased risk of dementia.

One of the key reasons proposed for the importance of mid-life hypertension on brain health is due to duration of exposure, as those with hypertension at age 50 are likely to be ‘exposed’ for longer.^3^ Direct assessment of duration of hypertensive status over many years is rare; many studies use a single measure of hypertension. An exception is the AGES study which used two measures of hypertension to examine the interaction between mid-life hypertension and late-life hypertension, separated by 26 years.^28^ They found that mid-life hypertension modifies the relationship between late-life blood pressure and cognitive performance.^28^ However, this study had no information on hypertension status between these two assessments and therefore the protective effects of later life hypertension may reflect a shorter duration of hypertension.^29^

Hypertension is currently classified by SBP or DBP ≥140/90 mmHg although in the majority of studies in this area a higher threshold (160/95 mmHg) has been used to define hypertension.^5^ However, age-specific treatment targets are being suggested for the reduction of CVD risk with a recent recommendation of SBP < 120 mmHg for those 50 years and younger.^16^ Trial data on the benefits of reducing SBP on cognitive outcomes is not conclusive; however, all the research in this domain is based on older adults and whether better control of blood pressure at younger ages modifies risk for dementia is unknown.

Hypertension is known to be associated with silent strokes, white matter lesions, and impairment of cerebral circulation leading to ischaemic injury.^3^ This suggests that part of the association of hypertension with dementia may not be explained by clinical CVD, a hypothesis that we were able to test using multi-state models which allow incorporation of both incident CVD and dementia over the follow-up. Our results show excess risk of dementia is also present in those free of CVD. These results suggest that subclinical or ‘silent’ vascular brain lesions (i.e. infarcts, microbleeds, white matter changes), which are common in those with hypertension may be involved in increased dementia risk in those with high blood pressure who do not have clinical CVD.^3^ Thus, cerebral small vessel disease is likely to be an important mechanism underlying the association of high blood pressure and cognitive dysfunction.

A limitation of the study is use of linkage to electronic health records for dementia ascertainment, a method that has high specificity but is likely to miss milder cases of dementia.^30^ There was no evidence in our data that hypertension affected age of dementia diagnosis, it was 75.3 and 75.2 years in groups defined by SBP ≥ 130 mmHg (yes/no) at age 50 (*P* = 0.85). Thus, any misclassification of dementia status is likely to be random, i.e. the probability of dementia status being misclassified is independent of hypertension at age 50. Under conditions of high specificity, the association between risk factor and outcome is unlikely to be biased by under-ascertainment of the outcome.^16^ Furthermore, under-ascertainment of dementia is unlikely to lead to the age-specific patterns observed in our study. Another limitation is that we were not able to examine whether the association of hypertension was stronger with Alzheimer’s or vascular dementia due to small numbers. Random measurement error in the blood pressure readings may have diluted associations but it is unlikely to lead to the pattern of results observed for SBP at 50, 60, and 70 years. Finally, residual confounding cannot be ruled out in observational studies but it is unlikely that bias would lead to the pattern of results observed in our study.

Strengths of the study are linked to the availability of repeat data on blood pressure and a long follow-up for dementia. Thus, both ‘timing’ and ‘duration’ which seem to be key to understanding the role of hypertension in dementia,^3^ could be examined. However, some misclassification is possible as blood pressure was assessed only every 4 years, and we did not have data on ambulatory blood pressure. A further strength is use of measured blood pressure rather than self-reported antihypertensive use to define hypertension, ensuring that reporting or non-compliance which can be considerable in observational studies do not bias our results. This approach also allows the development of clear neuroprotective guidelines, contributing to the debate on the correct target for blood pressure.^16^ Finally, the use of IPW ensures that missing data do not affect results, as they are known to be more common in those at greater risk of adverse health outcomes.^18^

## Conclusion

Hypertension is a known risk factor for CVD, renal failure, and premature mortality. It is also highly prevalent; number of persons with elevated SBP continues to increase globally and may affect dementia risk either directly or via processes related to CVD. Our study highlights the detrimental effects of mid-life hypertension, here at age 50 years, and increase in risk at levels below that used to treat SBP.

## Supplementary Material

Supplementary Data
