# Supplementary material for: Association between systolic blood pressure and dementia in the Whitehall II cohort study: role of age, duration, and threshold used to define hypertension
Source: Eur Heart J. 2018 Jun 12;39(33):3119–25. doi: 10.1093/eurheartj/ehy288 (PMC6122131; doi:10.1093/eurheartj/ehy288)
Supplement: Supplementary Data [file ehy288_supplementary_tables_and_figures.docx]

**Supplementary Tables and Figures**

**Table S1. Association between 5 categories of systolic blood pressure and incidence of dementia.**

**Table S2. Age & threshold of systolic/diastolic blood pressure: association between hypertension and incidence of dementia.**

**Table S3. Age & threshold of systolic blood pressure: association between hypertension (systolic blood pressure ≥130 mmHg OR Anti-hypertensive medication) and incidence of dementia.**

**Table S4. Estimation of trajectories of blood pressure: model fit statistics (group based trajectory models).**

**Table S5. Duration of hypertension** **(systolic blood pressure ≥130 mmHg OR Anti-hypertensive medication) trajectories with incidence of dementia.**

**Figure S1. Trajectory of global cognitive score in dementia cases in the years leading to dementia diagnosis and dementia free participants until end of follow-up.**

**Figure S2. Threshold: association of diastolic blood pressure at age 50 (Panel A), 60 (Panel B), and 70 years (Panel C) with dementia.**

**Figure S3. Trajectories of hypertension (data from 1985, 1991, 1997, 2003), defined using systolic blood pressure ≥130 mmHg.**

**Table S1. Association between 5 categories of systolic blood pressure**

**and incidence of dementia.**

|  | **N dementia / N total** | **HR* (95%CI)** |
| --- | --- | --- |
| **Systolic blood pressure at age 50 (mmHg)** | | |
| <110 | 56/1,735 | 1.00 |
| 110-119 | 91/2,272 | 1.15 (0.82, 1.61) |
| 120-129 | 81/2,159 | 1.02 (0.72, 1.44) |
| 130-139 | 83/1,420 | 1.49 (1.05, 2.11) |
| ≥140 | 74/1,053 | 1.61 (1.13, 2.29) |
| **Systolic blood pressure at age 60 (mmHg)** | | |
| <110 | 56/1,230 | 1.00 |
| 110-119 | 75/1,706 | 1.04 (0.72, 1.48) |
| 120-129 | 80/1,875 | 1.08 (0.76, 1.54) |
| 130-139 | 64/1,408 | 1.01 (0.70, 1.46) |
| ≥140 | 65/1,339 | 1.20 (0.83, 1.74) |
| **Systolic blood pressure at age 70 (mmHg)** | | |
| <110 | 35/558 | 1.00 |
| 110-119 | 34/905 | 0.56 (0.34, 0.93) |
| 120-129 | 52/1,231 | 0.76 (0.46, 1.24) |
| 130-139 | 55/1,028 | 0.83 (0.52, 1.34) |
| ≥140 | 69/1,267 | 0.77 (0.49, 1.21) |

* Analysis using inverse probability weighting in Cox regression, adjusted for age, sex, education, ethnicity, marital status, occupational position.

**Table S2. Age & threshold of systolic/diastolic blood pressure: association between hypertension and incidence of dementia.^a^**

|  |  | **Model 1** | **Model 2** | **Model 3** |
| --- | --- | --- | --- | --- |
|  | **N cases/N total** | **HR (95%CI)** | **HR (95%CI)** | **HR (95%CI)** |
| **Hypertension at age 50 Years (N=8,639)** | | | | |
| **Systolic blood pressure ≥140 mmHg OR Diastolic blood pressure ≥90 mmHg** | | | | |
| No | 287/7,046 | 1.00 | 1.00 | 1.00 |
| Yes | 98/1,593 | 1.34 (1.07, 1.69) | 1.35 (1.08, 1.70) | 1.26 (0.99, 1.59) |
| **Systolic blood pressure ≥130 mmHg OR Diastolic blood pressure ≥90 mmHg** | | | | |
| No | 222/5,984 | 1.00 | 1.00 | 1.00 |
| Yes | 163/2,655 | 1.45 (1.18, 1.78) | 1.45 (1.18, 1.78) | 1.37 (1.11, 1.69) |
| **Systolic blood pressure ≥120 mmHg OR Diastolic blood pressure ≥90 mmHg** | | | | |
| No | 147/3,985 | 1.00 | 1.00 | 1.00 |
| Yes | 238/4,654 | 1.19 (0.97, 1.47) | 1.18 (0.95, 1.45) | 1.09 (0.88, 1.35) |
| **Hypertension at age 60 Years (N=7,558)** | | | | |
| **Systolic blood pressure ≥140 mmHg OR Diastolic blood pressure ≥90 mmHg** | | | | |
| No | 254/5,979 | 1.00 | 1.00 | 1.00 |
| Yes | 86/1,579 | 1.18 (0.92, 1.52) | 1.17 (0.91, 1.51) | 1.15 (0.89, 1.49) |
| **Systolic blood pressure ≥130 mmHg OR Diastolic blood pressure ≥90 mmHg** | | | | |
| No | 202/4,743 | 1.00 | 1.00 | 1.00 |
| Yes | 138/2,815 | 1.11 (0.89, 1.39) | 1.11 (0.90, 1.40) | 1.08 (0.85, 1.36) |
| **Systolic blood pressure ≥120 mmHg OR Diastolic blood pressure ≥90 mmHg** | | | | |
| No | 131/2,929 | 1.00 | 1.00 | 1.00 |
| Yes | 209/4,629 | 1.06 (0.85, 1.33) | 1.07 (0.85, 1.34) | 1.05 (0.83, 1.33) |
| **Hypertension at age 70 Years (N=4,989)** | | | | |
| **Systolic blood pressure ≥140 mmHg OR Diastolic blood pressure ≥90 mmHg** | | | | |
| No | 175/3,696 | 1.00 | 1.00 | 1.00 |
| Yes | 70/1,293 | 0.97 (0.71, 1.33) | 0.96 (0.70, 1.32) | 1.02 (0.73, 1.42) |
| **Systolic blood pressure ≥130 mmHg OR Diastolic blood pressure ≥90 mmHg** | | | | |
| No | 121/ 2,687 | 1.00 | 1.00 | 1.00 |
| Yes | 124/2,302 | 1.06 (0.80, 1.40) | 1.05 (0.79, 1.41) | 1.14 (0.84, 1.53) |
| **Systolic blood pressure ≥120 mmHg OR Diastolic blood pressure ≥90 mmHg** | | | | |
| No | 69/1,463 | 1.00 | 1.00 | 1.00 |
| Yes | 176/3,526 | 1.07 (0.78, 1.46) | 1.06 (0.78, 1.45) | 1.19 (0.86, 1.64) |

^a^ Analysis using inverse probability weighting in Cox regression.

Model 1: Adjusted for age, sex, education, ethnicity, marital status, occupational position.

Model 2: Model 1 + smoking, alcohol consumption, fruit & vegetable consumption, physical activity.

Model 3: Model 2 + BMI, diabetes at start of follow-up + time-dependent cardiovascular disease (coronary heart disease, stroke), atrial fibrillation, heart failure and cardiovascular disease medication.

**Table S3. Age & threshold of systolic blood pressure: association between hypertension (high systolic blood pressure OR Anti-hypertensive medication) and incidence of dementia.^a^**

|  |  | **Model 1** | **Model 2** | **Model 3** |
| --- | --- | --- | --- | --- |
|  | **N cases/N total** | **HR (95%CI)** | **HR (95%CI)** | **HR (95%CI)** |
| **Hypertension at age 50 Years (N=8,639)** | | | | |
| **Systolic blood pressure ≥140 mmHg OR Anti-hypertensive medication** | | | | |
| No | 293/7,223 | 1.00 | 1.00 | 1.00 |
| Yes | 92/1,416 | 1.43 (1.13, 1.81) | 1.43 (1.13, 1.82) | 1.32 (1.03, 1.69) |
| **Systolic blood pressure ≥130 mmHg OR Anti-hypertensive medication** | | | | |
| No | 216/5,909 | 1.00 | 1.00 | 1.00 |
| Yes | 169//2,730 | 1.48 (1.21, 1.82) | 1.48 (1.21, 1,82) | 1.40 (1.14, 1.73) |
| **Systolic blood pressure ≥120 mmHg OR Anti-hypertensive medication** | | | | |
| No | 143/ 3,876 | 1.00 | 1.00 | 1.00 |
| Yes | 242/4,763 | 1.19 (0.96, 1.47) | 1.17 (0.95, 1.45) | 1.08 (0.87, 1.34) |
| **Hypertension at age 60 Years (N=7,558)** | | | | |
| **Systolic blood pressure ≥140 mmHg OR Anti-hypertensive medication** | | | | |
| No | 225/5,069 | 1.00 | 1.00 | 1.00 |
| Yes | 115//2,489 | 1.37 (1.09, 1.72) | 1.35 (1.07, 1.70) | 1.36 (1.07, 1.74) |
| **Systolic blood pressure ≥130 mmHg OR Anti-hypertensive medication** | | | | |
| No | 173/3,964 | 1.00 | 1.00 | 1.00 |
| Yes | 167/3,594 | 1.26 (1.02, 1.57) | 1.25 (1.01, 1.56) | 1.24 (0.98, 1.56) |
| **Systolic blood pressure ≥120 mmHg OR Anti-hypertensive medication** | | | | |
| No | 115/2,479 | 1.00 | 1.00 | 1.00 |
| Yes | 225/5,079 | 1.12 (0.89, 1.41) | 1.12 (0.90, 1.42) | 1.11 (0.87, 1.41) |
| **Hypertension at age 70 Years (N=4,989)** | | | | |
| **Systolic blood pressure ≥140 mmHg OR Anti-hypertensive medication** | | | | |
| No | 114/2,334 | 1.00 | 1.00 | 1.00 |
| Yes | 131/2,655 | 1.11 (0.84, 1.47) | 1.08 (0.81, 1.43) | 1.18 (0.85, 1.66) |
| **Systolic blood pressure ≥130 mmHg OR Anti-hypertensive medication** | | | | |
| No | 78/ 1,719 | 1.00 | 1.00 | 1.00 |
| Yes | 167/3,270 | 1.18 (0.87, 1.60) | 1.15 (0.85, 1.56) | 1.25 (0.89, 1.76) |
| **Systolic blood pressure ≥120 mmHg OR Anti-hypertensive medication** | | | | |
| No | 47/952 | 1.00 | 1.00 | 1.00 |
| Yes | 198/4,037 | 1.16 (0.82, 1.64) | 1.13 (0.80, 1.60) | 1.23 (0.84, 1.81) |

^a^ Analysis using inverse probability weighting in Cox regression.

Model 1: Adjusted for age, sex, education, ethnicity, marital status, occupational position.

Model 2: Model 1 + smoking, alcohol consumption, fruit & vegetable consumption, physical activity.

Model 3: Model 2 + BMI, diabetes at start of follow-up + time-dependent cardiovascular disease (coronary heart disease, stroke), atrial fibrillation, heart failure and cardiovascular disease medication.

**Table S4. Estimation of trajectories of blood pressure: model fit statistics (group based trajectory models).**

| **Group**  **size ^a^** | **Trajectory**  **shape ^b^** | **Allocated**  **Group membership** | **BIC**  **(sample)^c^** | **Average Posterior**  **Probabilities^d^** | **AIC ^e^** | **Odds correct**  **classification ^f^** |
| --- | --- | --- | --- | --- | --- | --- |
| 1 | 2 | 100% | -17795.34 |  | -17784.80 |  |
| 2 | 2  2 | 56.7%  43.3% | -15992.55 | 0.92  0.89 | -15967.96 | 10.6  8.8 |
| 3 | 2  2  2 | 61.8%  5.4%  32.8% | -15835.09 | 0.91  0.64  0.90 | -15796.44 | 6.7  27.9  17.5 |
| 4 | 2  2  2  2 | 33.9%  22.7%  16.6%  26.9% | -15838.96 | 0.75  0.69  0.71  0.84 | -15786.26 | 5.8  7.5  12.0  14.2 |
| 3 | 0  2  2 | 51.4%  16.0%  32.6% | -15873.28 | 0.87  0.68  0.94 | -15841.67 | 7.0  12.0  27.9 |
| 3 | 1  1  1 | 47.7%  18.9%  33.8% | -15872.01 | 0.76  0.83  0.89 | -15843.91 | 12.6  15.0  5.2 |
| 3 | 1  1  2 | 48.8%  19.1%  32.2% | -15851.15 | 0.76  0.82  0.91 | -15819.53 | 5.2  11.7  19.6 |
| **3** | **1**  **2**  **2** | **48.8%**  **18.6%**  **32.7%** | **-15844.26** | **0.76**  **0.78**  **0.96** | **-15809.13** | **5.0**  **11.2**  **40.9** |

^a^ Number of trajectory groups estimated (N=8,315); ^b^ Polynomial function of time (0 intercept only, 1 linear, 2 quadratic);

^c^ Bayesian Information Criterion (BIC), a difference of 10 is strong evidence that the model with the lowest BIC (compared to null) has best fit;

^d^ Posterior probabilities of group membership for individuals assigned to each group, an average > 0.7 demonstrates good classification accuracy;

^e^ Akaike Information Criterion (AIC); ^f^ Odds of correct classification based on posterior probabilities and group membership, minimum threshold of 5.

Model selected based on fulfilment of criteria d & f and evidence of improved fit using lowest BIC/AIC score.

**Table S5. Duration of hypertension** **(systolic blood pressure ≥130 mmHg OR Anti-hypertensive medication) trajectories^a^ with incidence of dementia.^b^**

| **N=** **8,313** |  | **Model 1** | **Model 2** | **Model 3** |
| --- | --- | --- | --- | --- |
|  | **N cases/N total** | **HR (95% CI)** | **HR (95% CI)** | **HR (95% CI)** |
| **Hypertension trajectories (data from 1985, 1991, 1997, 2003)** | | | | |
| Group 1: Low | 109/3,607 | 1.00 | 1.00 | 1.00 |
| Group 2: Increasing | 70/1,686 | 1.12 (0.82, 1.51) | 1.13 (0.83, 1.54) | 1.18 (0.85, 1.62) |
| Group 3: High | 179/3,020 | 1.37 (1.06, 1.76) | 1.36 (1.06, 1.76) | 1.38 (1.06, 1.81) |

^a^ The trajectories over a mean 16 year period were again determined using a group based trajectory method (1,2,2) as described previously.

^b^ Analysis using inverse probability weighting in Cox regression.

Model 1: Adjusted for age, sex, education, ethnicity, marital status, occupational position.

Model 2: Model 1 + smoking, alcohol consumption, fruit & vegetable consumption, physical activity.

Model 3: Model 2 + BMI, diabetes at start of follow-up + time-dependent cardiovascular disease (coronary heart disease, stroke), atrial fibrillation, heart failure and cardiovascular disease medication.

**Figure S1. Trajectory of global cognitive score** ^a^ **in dementia cases in the years leading to dementia diagnosis and dementia free participants until end of follow-up.**

|  | **Number of observations in the analysis** | | | | | |
| --- | --- | --- | --- | --- | --- | --- |
| **Years** | | **-20 to -16** | **-16 to -12** | **-12 to-8** | **-8 to-4** | **-4 to 0** |
| **Dementia free (N=7237)** | | 5136 | 5693 | 5130 | 5788 | 5768 |
| **Dementia cases (N=291)** | | 88 | 125 | 167 | 177 | 145 |

^a^ Composed of tests of memory, reasoning, phonemic and semantic fluency administered to the participants in 1997, 2003, 2007, 2012, and 2015.

**Figure S2. Threshold: association of diastolic blood pressure^a,b^ at age 50 (Panel A), 60 (Panel B), and 70 years (Panel C) with dementia.**


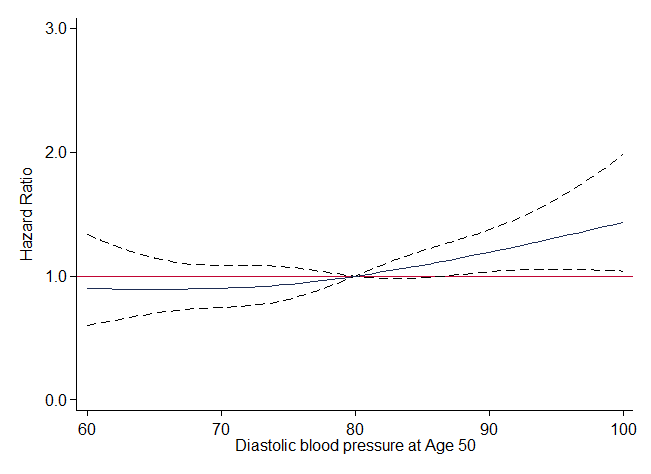


**Panel A**


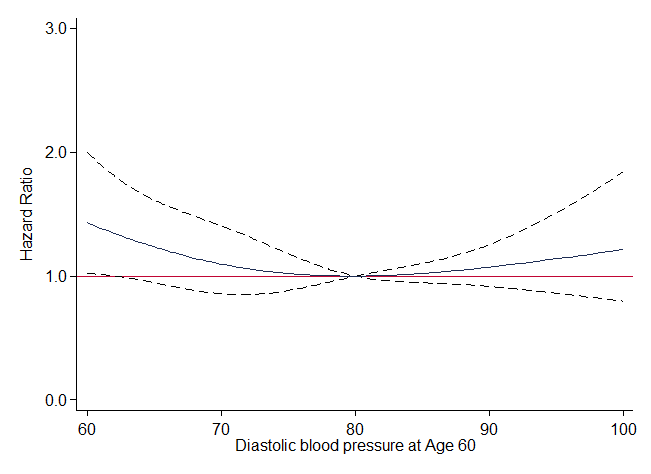


**Panel B**


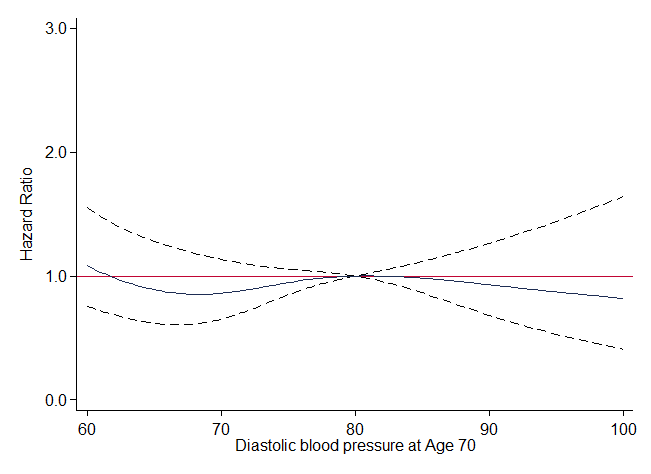


**Panel C**

^a^ Diastolic blood pressure was modelled by both-tail restricted cubic splines with four age-specific Harrell knots in a Cox regression model adjusted for age, sex, education, ethnicity, marital status, and occupational position. ^b^ The reference value for calculation of HRs is diastolic blood pressure 80 mmHg.

**Figure S3. Trajectories of hypertension (data from 1985, 1991, 1997, 2003), defined using systolic blood pressure ≥130 mmHg.^a^**


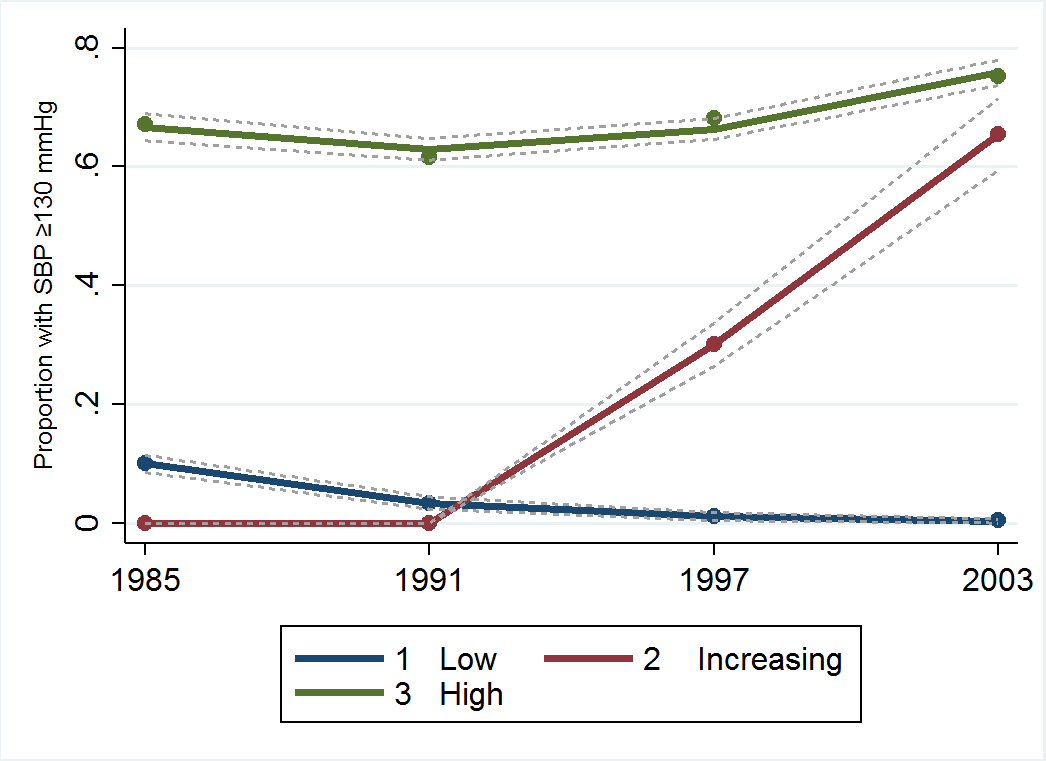


^a^ Three group solution (1,2,2). N= 8,313 (excluding those who had dementia or who had died before 2003 and excluding those with only one blood pressure assessment between 1985 and 2003).
